# A Computational Framework for Controlling the Self-Restorative Brain Based on the Free Energy and Degeneracy Principles

**DOI:** 10.3389/fncom.2021.590019

**Published:** 2021-04-14

**Authors:** Hae-Jeong Park, Jiyoung Kang

**Affiliations:** ^1^Center for Systems and Translational Brain Science, Institute of Human Complexity and Systems Science, Yonsei University, Seoul, South Korea; ^2^Department of Nuclear Medicine, Yonsei University College of Medicine, Seoul, South Korea; ^3^Department of Psychiatry, Yonsei University College of Medicine, Seoul, South Korea; ^4^Brain Korea 21 Project, Graduate School of Medical Science, Yonsei University College of Medicine, Seoul, South Korea; ^5^Department of Cognitive Science, Yonsei University, Seoul, South Korea

**Keywords:** free energy principle, resting state, brain dynamics, energy landscape, self-restoration, maximum entropy model, degeneracy

## Abstract

The brain is a non-linear dynamical system with a self-restoration process, which protects itself from external damage but is often a bottleneck for clinical treatment. To treat the brain to induce the desired functionality, formulation of a self-restoration process is necessary for optimal brain control. This study proposes a computational model for the brain's self-restoration process following the free-energy and degeneracy principles. Based on this model, a computational framework for brain control is established. We posited that the pre-treatment brain circuit has long been configured in response to the environmental (the other neural populations') demands on the circuit. Since the demands persist even after treatment, the treated circuit's response to the demand may gradually approximate the pre-treatment functionality. In this framework, an energy landscape of regional activities, estimated from resting-state endogenous activities by a pairwise maximum entropy model, is used to represent the pre-treatment functionality. The approximation of the pre-treatment functionality occurs via reconfiguration of interactions among neural populations within the treated circuit. To establish the current framework's construct validity, we conducted various simulations. The simulations suggested that brain control should include the self-restoration process, without which the treatment was not optimal. We also presented simulations for optimizing repetitive treatments and optimal timing of the treatment. These results suggest a plausibility of the current framework in controlling the non-linear dynamical brain with a self-restoration process.

## Introduction

The goal of clinical treatment for the brain is to modify the brain circuit to yield a desirable brain function. Since the brain is a complex non-linear dynamic system, clinical treatment can be considered a control problem. For the clinical treatment to the human brain, various methods have been developed, such as thermal ablation with the high intensity focused ultrasound (Park et al., 2017), deep brain stimulation (DBS) (Park et al., 2015), vagus nerve stimulation (Yu et al., 2018), and transcranial magnetic stimulation (TMS) (Park et al., 2013; Kar, 2019) as well as conventional medications and traditional surgical operations (Schreglmann et al., 2018). Despite the remarkable advancement of these therapeutic techniques, the optimal control of the brain by treatments has many practical challenges due to the complexity of the brain and ethical issues. Since various experiments are not allowed in the human brain, establishing optimal control procedures for the brain is very slow and limited. Therefore, optimal brain control remains mostly theoretical and based on computational models.

The brain control studies with computational models have been conducted mainly in two approaches: characterization of the network controllability and prediction of the network behavior based on the state dynamic equation of the brain. To characterize the brain network, the controllability of a brain system has been evaluated in the graph-theoretic perspective (Liu et al., 2011; Gu et al., 2017; Tang et al., 2017; Cornblath et al., 2019; Lee et al., 2019; Stiso et al., 2019; Karrer et al., 2020). This approach optimizes input signals to increase or decrease activity at some brain network nodes to induce the desired brain activity at all the brain nodes. The other approach is to predict a brain system's behavior by altering some nodes or edges of the brain. In this approach, the optimal control is determined by evaluating the outcome after removing nodes or edges or changing the system's parameters in the virtual brain model (Falcon et al., 2016; Jirsa et al., 2017; Proix et al., 2017; An et al., 2019; Olmi et al., 2019). Those two types of computational approaches on brain control have primarily focused on the immediate changes in the brain network's activity or function. Those studies, however, did not consider the fact that the brain has self-restorative plasticity, making the system resilient to external treatments or perturbations.

Self-restoration capacity in the brain has been found after damage or stress via neural, molecular, and hormonal mechanisms (Russo et al., 2012; King, 2016; Murrough and Russo, 2019). At the macroscopic level, the self-restoration process toward the initial functionality has widely been reported in clinical neuroscience, for e.g., functional recovery after stroke (Murphy and Corbett, 2009; Malone and Felling, 2020), recovery of the language capacity (Saur et al., 2006), recovery of the vision after surgery (Mikellidou et al., 2019). This self-restorative property of the brain is advantageous in protecting the brain after various external attacks (Glassman, 1987). In terms of clinical treatment, however, this self-restoration process is a bottleneck as it tends to recover the initial abnormal functionality, acting against the aim of any treatment. Examples of this anti-treatment action can be found with neurological or antipsychotic medication (Abbott, 2010) showing drug resistance in most brain disease such as schizophrenia (Potkin et al., 2020), depression (Bennabi et al., 2019), Parkinson's diseases (Vorovenci et al., 2016), and epilepsy (Lee et al., 2017). Goellner et al. (2013) showed that the late seizure recurrence after temporal lobe epilepsy surgery was as much as 48.9%. Despite the anatomical alteration by resection, the treated brains, initially showing free or reduction of abnormal function (seizure behavior) after surgical dissection, returned to the initial state of abnormal functionality in a certain period after treatment.

In this respect, the brain's self-restoration process may well be included as an essential part of the computational model of brain control. Despite the criticality of the brain's self-restoration process, research that has a self-restoration process in the control problem is hardly found. This may be partly attributable to the difficulty in defining the driving force of the self-restoration process and its mathematical formulation. In contrast to the microscopic level, where the mechanism of the self-recovery process has actively been researched in terms of neurogenesis (e.g., Mattson, 2008), a systematic understanding of the self-restoration process at the macroscopic level is still lacking. Are there any principles that we may refer to formulate the brain's self-restoration process at the macroscopic level?

In the current study, as an extension of our previous study (Kang et al., 2021), we propose a computational framework for controlling the self-restorative brain by formulating the driving force of self-restoration based on the free-energy principle (Friston et al., 2006; Friston, 2010) and the degeneracy nature of a non-linear complex system (Glassman, 1987). According to the free-energy principle, the brain acts based on a model established to minimize long-term average surprise from the external environment (Friston et al., 2006; Friston, 2010). The brain network and its behavior can be considered a result of long-term adjustment to meet environmental demands (Park and Friston, 2013). For a neural population at any level of the information hierarchy, neural populations that send signals to and receive signals from the neural population are environment to the neural population. For a neural circuit of the brain, the circuit's environment can involve lower-level and higher-level neural populations, affecting the neural circuit by sensation from the lower-level neural populations and regulation from the higher-level neural populations (Friston, 2008). The long-term demands of the environment to a neural circuit or system can be represented by the statistics of bottom-up (from the lower-level neural populations) and top-down (from the higher-level neural populations) signals. Although any clinical treatment may alter a neural circuit, the circuit's environmental demands persist and do not change rapidly even after alteration in the circuitry. Since the functionality before treatment has been developed as an optimal solution to environmental demands, we posit that the altered neural circuit gradually approximates the pretreated neural circuit's functionality while adjusting itself to meet the environmental demands after treatment.

In this framework, the pre-treatment state the brain circuit tends to recover is not the same circuit, but the functionality that the circuit has established to satisfy the external demands. In most cases, the functionality for ongoing environmental demands has to be approximated via reallocation of the reduced circuit resources after treatment (e.g., after removing a node of the circuit). In this respect, the recovery of functionality via an altered circuit can be referred to as a well-known property of the complex biological system called “degeneracy” (Glassman, 1987; Edelman and Gally, 2001). Degeneracy of a system indicates a function (or behavior) can be implemented with different network configurations (Friston and Price, 2003). Degeneracy of the self-reorganizing biological system is essential to manage and protect its functionality from damage (Marder and Goaillard, 2006; Marder et al., 2017). This study is based on the degeneracy principle as it focuses on the restoration of the pre-treatment functionality, not the pre-treatment circuit.

The final component of the current framework for brain control is to define the pre-treatment functionality of the brain. As a representation of the brain's functionality, we used the non-linear dynamics of regional activity while the brain is at rest (called resting-state). A strong relationship exists between the resting-state connectivity (or distributed patterns of endogenous activity) with task-related brain activation or connectivity (Biswal et al., 1995; Smith et al., 2009; Cole et al., 2014, 2016; Krienen et al., 2014; Park et al., 2014; Yeo et al., 2015; Tavor et al., 2016; Jung et al., 2018). Fox and Raichle (2007) argued that resting-state connectivity might serve as a potential scaffold that supports diverse configurations subserving the functional elements of a given task (Fox and Raichle, 2007). In this respect, a stabilized brain network's resting-state dynamics were considered to summarize the environmental demands established by long-term interactions with the environment.

The resting-state brain network may behave as a non-linear dynamical system with its microstate (defined by distributed regional activity pattern) transitioning over the energy landscape of multistable microstates (attractors) (Freyer et al., 2011, 2012; Rabinovich and Varona, 2011; Deco and Jirsa, 2012; Kelso, 2012; Cabral et al., 2014; Tognoli and Kelso, 2014; Deco et al., 2015; Breakspear, 2017). The non-linear dynamics of the brain circuit can be modeled in terms of non-linear interactions among nodes in the network using a pairwise maximum entropy model (MEM) (Watanabe et al., 2013, 2014a,b,c; Kang et al., 2017, 2019; Ezaki et al., 2018; Gu et al., 2018). From the pairwise MEM, we can infer the probability distribution of each microstate (an activation pattern), the microstates' energy landscape. In the energy landscape, a microstate's energy is the minus (scaled) log of its probability of occurrence. In this model, the microstate dynamics (represented in microstates' energy landscape) are emergent from the underlying complex network (or circuitry). They are considered to represent the gross functionality of a brain.

In summary, our proposal for the recovery process can be explained by the free energy principle to satisfy environmental demands by reconfiguring the remained resources after treatment according to the degeneracy principle of the complex brain. Utilizing this self-restoration model, we could develop a strategy to identify the optimal treatment target region (nodes or edges in the network) and the amount of treatment strength within a source system to be treated (e.g., a disease system) to induce microstate dynamics of the desired goal system (a healthy system). We call this procedure optimal brain control throughout the paper. In the conventional control theory problems, control signals are inputs to the system to achieve the desired system's state without changing the system parameters. Meanwhile, the optimal brain control in this study refers to adjusting the source system's model parameters to approximate the desired functionality of the goal system. In this study based on the pairwise MEM, the model parameters include the sensitivity of a brain region and interaction among brain regions, which indicate neurobiological connectivity or synaptic efficacy that modulate the input and output relationship, i.e., the functionality of the brain.

We used the term “optimal brain control” in consideration of clinical treatment settings that call for optimal selection of treatment target gray matter regions (nodes) or white matter regions (edges) and treatment strength, within limited access to the brain circuit at a time. For example, the circuit that generates epileptic seizures is the source system, and the goal system is a healthy functioning state without a seizure. The treatment target can be multiple nodes in the medication. For example, lorazepam enhances the effect of the inhibitory neurotransmitter gamma-aminobutyric acid (GABA) receptors distributed in multiple brain areas. The target can be a single node by temporal lobectomy, which removes a part of the anterior temporal lobe. Callosotomy, which dissects interhemispheric fibers, is an example of targeting edges in the network.

The current paper is composed in the following order. It begins with a mathematical description of pairwise MEM and its energy landscape analysis. We then formulated a self-restoration process and optimal control in a non-linear dynamical system. Based on this formulation, we present diverse simulations to illustrate the self-restoration process and show the effect of modeling the self-restoration process in brain control. We also present simulations for optimizing repetitive treatment strategy and its timing in consideration of the clinical practice, where any treatment is highly restricted. Using these simulations, we expected to show the construct validity of the current framework in the brain's control.

## Background

### Dynamic Properties of the Brain Using Pairwise Maximum Entropy Model

To define the dynamic properties of a system, we used the energy landscape of microstate established on pairwise MEM. Here, we briefly explain the pairwise MEM model. The details for deriving the MEM of the resting-state functional magnetic resonance imaging (rsfMRI) can be found elsewhere (Watanabe et al., 2013, 2014a; Kang et al., 2017, 2019).

In the pairwise MEM model of a brain with N regions, a brain state *V*_*k*_, is defined as an *N*-dimensional binary vector;

(1) $$\begin{array}{l} V_{k}=\left(\sigma_{1},...,\sigma_{N}\right), \end{array}$$

where σ_*i*_ = 1 for an activated state and σ_*i*_ = 0 for an inactivated state at region *i*. Thus, totally 2^*N*^ states exist. An energy *E* (*V*_*k*_) of a state *V*_*k*_ is defined as

(2) $$\begin{array}{l} E\left(V_{k}\right)=-\overset{N}{\underset{i=1}{\sum }}H_{i}\sigma_{i}\left(V_{k}\right)-\overset{N-1}{\underset{i=1}{\sum }}\overset{N}{\underset{j=i+1}{\sum }}J_{ij}\sigma_{i}\left(V_{k}\right)\sigma_{j}\left(V_{k}\right), \end{array}$$

where *H*_*i*_ and *J*_*ij*_ are model parameters that represent weights for independent activation of region *i* and pairwise interaction (coactivation) between regions *i* and *j*, respectively. For simplicity, we used *A* = {*H*_*i*_, *J*_*ij*_}|_*i* = 1, ⋯ , *N, j* = 1, ⋯ , *N*_ to express all the model parameters. These model parameters were estimated using the maximum likelihood estimation approach. For a detailed mathematical review, see Yeh et al. (2010).

The probability of a state *V*_*k*_ is given by the Boltzmann distribution *p* (*V*_*k*_),

(3) $$\begin{array}{l} p\left(V_{k}\right)=\frac{\exp\left(-E\left(V_{k}\right)\right)}{\sum_{j=1}^{2^{N}}\exp\left(-E\left(V_{j}\right)\right)}. \end{array}$$

To analyze the energy landscape of state dynamics, we defined local minima (attractors, *LM*) and occupation time ratio of each local minimum (*OCR*(*LM*_*i*_)) as below.

#### Local Minimum

A local minimum (*LM*_*i*_) of an energy landscape of a system with parameters A is a state with lower energy than its neighbor states. Neighbor states are defined as states that differ from each other by only one element (one region) of the activation state.

#### Occupation Time Ratio

*OCR*(*LM*_*i*_): Occupation time ratio of *LM*_*i*_ is the sum of probabilities of all states in the basin region of *LM*_*i*_. The basin region of *LM*_*i*_ is the set of states that belong to the *LM*_*i*_. To determine whether a state belongs to the *LM*_*i*_, each element of the state is gradually changed along the energy gradient until it reaches one of the local minima.

### Functional Distance Between Energy Landscapes

To measure the functional distance between the target and source systems, *A*^*t*^ and *A*^*s*^, in the energy landscape, we defined a distance function between states in terms of dynamic properties (energy landscape) of the two systems, governed by the system's network parameters.

To focus on the functional distance between major attractors and their properties in the optimal treatment, we use a partial Kullback–Leibler (KL)-divergence defined as follows.

(4) $$\begin{array}{l} D\left(A^{t},A^{s}\right)=\underset{k\in \mathbb{R}}{\sum }p\left(V_{k}|A^{t}\right)\ln\frac{p\left(V_{k}|A^{t}\right)}{p\left(V_{k}|A^{s}\right)}, \end{array}$$

where ℝ represents a set of states that belong to basin regions of major attractors.

We also defined the similarity between two systems in terms of system parameters by the root-mean-square deviation (RMSD) of the two systems' parameter vectors.

(5) $$\begin{array}{l} RMSD\left(A^{t},A^{s}\right)=\left\|A^{t}-A^{s}\right\|. \end{array}$$

### Recovery Process

We modeled the recovery process based on three assumptions: (1) recovery occurs by adjusting the network connectivity (interactions) of the neighbors of the treated node or edge; (2) adjustment of connectivity is performed within a range of its flexibility, and (3) recovery occurs to meet the external demands, which were represented in the state dynamics of the pretreated stabilized system.

The treatment at region *m* (a node or an edge, for simplicity, we call it “region”) is denoted by changing the element $A_{m}^{p}\;$in the pretreated network parameters *A*^*p*^ with

(6) $$\begin{array}{l} A_{m}^{tr}⇐A_{m}^{p}+\alpha \end{array}$$

where α is the amount of treatment. The system parameters right after treatment can be expressed as $A^{t}=\left\{A_{m}^{tr},A_{\backslash m}^{p}\right\}$, where *m* and \*m* represent the treated and untreated regions, respectively (Figure 2D). *m* can be multiple nodes or edges. In this theoretical study, we assumed that we know how to achieve the desired level α and achieve the desired parameter $A_{m}^{tr}$. The treated state *A*^*t*^ of a system is the starting point of the recovery ${A_{0}}^{r}$.

The system proceeds with its recovery to minimize the functional distance between state dynamics before treatment and recovery (Figure 2B). When we decompose the network elements (nodes or edges) into recovery regions (strongly connected neighbors of the treated node or edge), ℝ_*m*_, and unchanged (weakly or unconnected) regions, \ℝ_*m*_, for a treated region (node or edge) *m* with a treatment strength $A_{m}^{tr}$, the network state of the treated system just after treatment, $A^{t}=\left\{A_{m}^{tr},A_{\backslash m}^{p}\right\}$, can be written as $A^{t}=\left\{A_{{\mathbb{R}}_{m}}^{t},A_{\backslash {\mathbb{R}}_{m}}^{t}\right\}$. The recovery then begins from *A*^*t*^ and the recovery regions ℝ_*m*_ cooperate to find an optimal parameter set $A_{{\mathbb{R}}_{m}}^{*}\;$within a constrained bound ℂ to return to the pre-treatment state *A*^*p*^. This recovery process can be written as below:

(7) $$\begin{array}{l} \;A_{{\mathbb{R}}_{m}}^{*}=\arg\underset{A_{{\mathbb{R}}_{m}}^{t^{\prime }},|A_{{\mathbb{R}}_{m}}^{t^{\prime }}|\leq \mathbb{C}}{\min}\;D_{r}\left(A^{p},A^{t^{\prime }}|A_{{\mathbb{R}}_{m}}^{t^{\prime }}\right)\\ \;A^{t^{\prime }}=\left\{A_{{\mathbb{R}}_{m}}^{t^{\prime }},A_{\backslash {\mathbb{R}}_{m}}^{t}\right\},A^{r}=\left\{A_{{\mathbb{R}}_{m}}^{*},A_{\backslash {\mathbb{R}}_{m}}^{t}\;\right\}, \end{array}$$

where $D_{r}$ indicates the distance function between the stabilized state before treatment *A*^*p*^ and a plausible treatment solution *A*^*t*′^ by adjusting parameters $A_{{\mathbb{R}}_{m}}^{t^{\prime }}$in the recovery region ℝ_*m*_ while keeping the other region \ℝ_*m*_ unchanged after treatment *A*^*t*^. The final recovered state *A*^*r*^ is composed of the optimal parameter set within the recovered regions $A_{{\mathbb{R}}_{m}}^{*}$ and the unchanged regions of the treated system $A_{\backslash {\mathbb{R}}_{m}}^{t}.$ Considering the limited capacity of the biological change, we restricted maximum changes at the recovered regions $A_{{\mathbb{R}}_{m}}^{*}$ to be <20 % of those of the previous step.

In this study, we define the pre-treatment network parameter *A*^*p*^ as a stable state after a long period of adaptation to the environment. The optimally recovered state *A*^*r*^ can be a new pre-treatment network state *A*^*p*^ for a subsequent treatment, after stabilization, e.g., ${A_{\infty }}^{r}=A^{p}$, where ∞ indicates a sufficient time for stabilization. Since the treated system may not revert completely to the pre-treatment network state by utilizing the constrained resources of the recovery regions, *A*^*p*^ is a function of trial number or time, moving toward the target system over a very long time scale. It should also be noted that a subsequent treatment can be applied to a system before the system is stabilized. We refer to treatment before stabilization as the treatment at the transient network stage. We considered that the transient state does not satisfy the environment's demands. In this case, the pre-treatment network parameter *A*^*p*^ was not updated with the state right before treatment, but instead referred to the recent stabilized state. We utilized functional distance $D_{r}$ to generate similar dynamics instead of generating similar network parameters for the recovering and initial systems.

The reorganization is performed by modifying the network parameters of neighbors ℝ_*m*_ but within a plausible range of each parameter (connectivity)'s flexibility. In Eq. 7, we denote this with $|A_{{\mathbb{R}}_{m}}^{t^{\prime }}|\leq \mathbb{C}$, indicating plausible parameters within a constrained bound ℂ. The amount of change at each node or edge in the recovery regions can be defined proportionally to the treated system's baseline (pretreated state). For the treated region *m*, we can assign less flexibility than for neighbors or assign inflexibility (i.e., no change) after treatment during the recovery process.

To measure effects of a treatment on brain dynamics, we define a recovery capacity as the difference between functional distance (partial KL divergence) of the treated state *A*^*t*^ and pretreated state *A*^*p*^, *D* (*A*^*p*^, *A*^*t*^), and the functional distance between the recovered state *A*^*r*^ and pretreated state *A*^*p*^, *D* (^*p*^, *A*^*r*^) as follows,

$$\begin{array}{l} \text{Recovery capacity}=△D=D\left(A^{p},A^{t}\;\right)-D\left(A^{p},A^{r}\;\right). \end{array}$$

### Optimal Control

A self-restorative system *A*^*s*^ is decomposed into the region that requires treatment *m* and the unaffected (untreated) region \*m* and is represented as $A^{s}=\left\{A_{m}^{s},A_{\backslash m}^{s}\right\}$. When a target system *A*^*g*^ is given as the goal to achieve for a source system *A*^*s*^, the optimal treatment is to find a region *m* and its treatment level $A_{m}^{t*}$ to minimize the distance function D between the goal system *A*^*g*^ and recovered system *A*^*s*′*r*^ that develops following the self-restoration process of the source system *A*^*s*^ in response to the treatment. To differentiate this from the distance function D between *A*^*g*^ and *A*^*s*′*r*^, we use $D^{+}$ to indicate the functional distance D between *A*^*g*^ and *A*^*s*^. The optimal control is defined as below,

(8) $$A_{m}^{t*}=\arg\;\min\;D^{+}\left(A^{g},A^{s^{\prime }}|A_{m^{\prime }}^{s^{\prime }}\right)$$

(9) $$\begin{array}{l} =\arg\;\min\;D\;\left(A^{g},A^{s^{\prime }r}|A_{m^{\prime }}^{s^{\prime }}\right), \end{array}$$

$$\begin{array}{l} \;A^{s^{\prime }}=\left\{A_{m^{\prime }}^{s^{\prime }},A_{\backslash m^{\prime }}^{s}\right\},\\ A^{s^{\prime }r}=\left\{A_{{\mathbb{R}}_{m^{\prime }}}^{s^{\prime }{}^{*}},A_{\backslash {\mathbb{R}}_{m^{\prime }}}^{s^{\prime }}\right\},\\ \;A^{t}=\left\{A_{m}^{t*},A_{\backslash m}^{s}\right\}, \end{array}$$

where the recovered system *A*^*s*′*r*^ is achieved following a self-restoration process after changes in the neighbors ${\mathbb{R}}_{m^{\prime }}\;$of the treated region *m*′, according to Equation 7. The optimal control is conducted by searching for the best solution to achieve the goal system's dynamics by adjusting the parameter $A_{m^{\prime }}^{s^{\prime }}$ in the treated region *m*′ while maintaining the other parameters $A_{\backslash m^{\prime }}^{s}\;$in untreated regions *m*′ unchanged. The final treated system *A*^*t*^ is composed of the optimal treatment region with its strength $A_{m}^{t*}$ and the unaffected parameters of the treated system $A_{\backslash m}^{s}.$

Note that the distance function D is defined in functional space (between energy landscapes), not in parameter space. In other words, D indicates a distance between the source dynamics that emerge from the source system with a parameter *A*^*s*^, and the target dynamics that emerge from the goal system with a parameter *A*^*g*^. From the perspective of degeneracy, the minimal distance function D in the dynamics space does not necessarily indicate the closeness in the network parameter space. Even though the two parameter sets, *A*^*g*^ and *A*^*s*^, are distant in the parameter space, they can be close in the dynamics space.

### Strategy for Iterative Optimal Treatment

**Figure 1:**
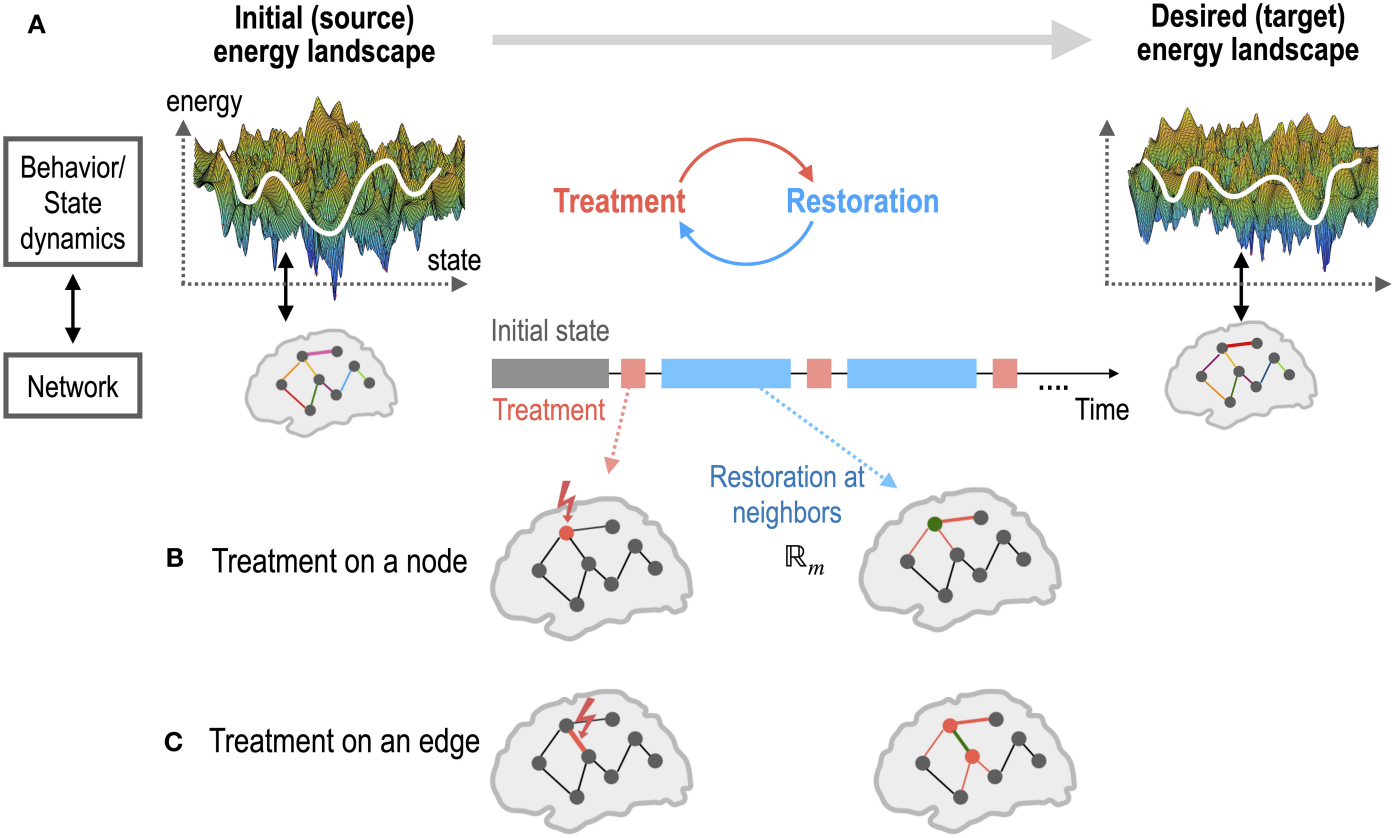
An illustration of the brain control for a self-restorative brain. **(A)** The state dynamics (or behaviors) of a source system and a desired target system emerge from the networks of the two systems. The state dynamics of a system is represented in the energy landscape, where the log of the inverse probability of each state (distributed brain activation) is defined as energy. The treatment on a network node or edge of the source system is determined to generate dynamics of the desired target system. Restoration is assumed to occur in the neighbors of the treated node **(B)** or edge **(C)**. Optimal control is the recursive procedure of treatment and restoration steps to achieve the desired dynamics of the goal system.

**Figure 2:**
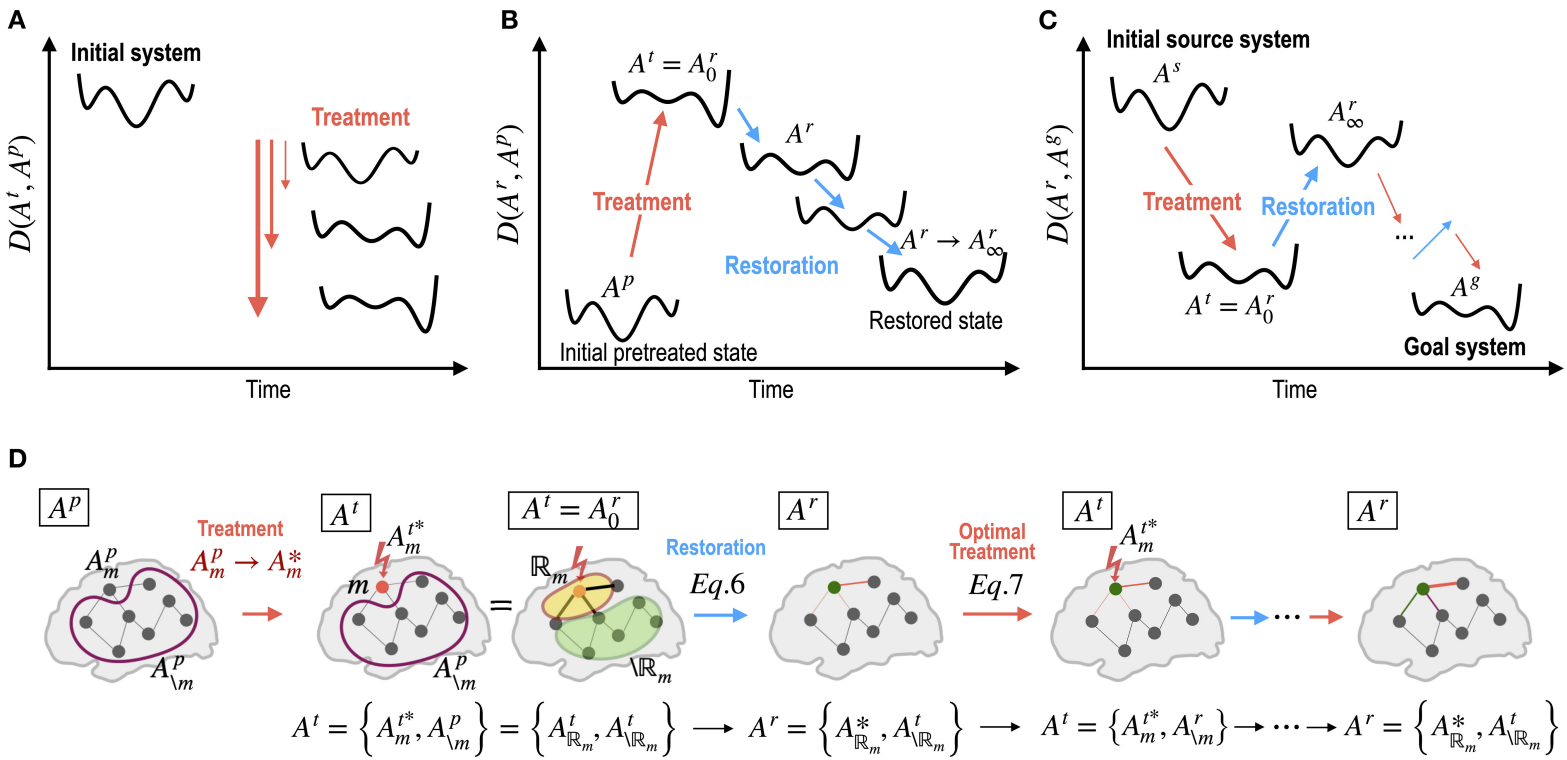
Dynamics of energy landscapes (state dynamics) by treatment in the network parameters and by self-restoration processes. **(A)** Different treatments on the network alter the energy landscape of the system non-linearly. **(B)** The brain has a tendency to revert to its initial state before treatment via non-linear state transitions. **(C)** Optimal treatment to induce desired behaviors (dynamics) can be achieved by iterative treatment and self-restoration via an optimal adjustment in the network parameters. **(D)** Notations for iterative procedures in the optimal control of the brain are explained. **A**^**p**^, **A**^**t**^, and **A**^**r**^ indicate system (network) parameters for the pretreated state, treated state, and self-recovery state; ${\text{A}}_{\text{m}}^{\text{t}*}$: optimal strength of a treated node or edge (m); ${\text{A}}_{\backslash \text{m}}^{\text{p}}$: the pre-treatment parameters of untreated node or edge (\m); ${\text{A}}_{{\mathbb{R}}_{\text{m}}}^{*}$: optimal parameters within the recovered regions ℝ_**m**_; ${\text{A}}_{\backslash {\mathbb{R}}_{\text{m}}}^{\text{t}}$: parameters of the unchanged regions in the recovery process. See the Method section for details.

Optimal treatment is a recursive procedure between treatment planning by the operator and the restoration process in the treated system (Figures 1, 2C). The target region (node and edge) to be treated and the strength of treatment was chosen using a grid search algorithm in this study. In practice, the treatment to the system was performed by altering the MEM parameter *A*_*i*_ (an activity of a region *H*_*i*_ or a pairwise interaction *J*_*ij*_) by an amount of α. We assumed that only neighboring nodes and edges of the treated node participate in the recovery process to return the brain dynamics to the pre-treatment state (Figures 1B,C). The treatment strength induces changes in the energy landscape in a non-linear manner (Figure 2A). When a node is altered (i.e., *H*_*i*_ is changed), edges that are strongly connected with the node (Figure 1B) undergo self-restoration steps, gradually changing the energy landscape (Figure 2B). When an edge is treated (i.e., *J*_*ij*_ is selected for treatment), two nodes that are connected with the treated edge and the strongly connected edges of the two nodes undergo self-restoration (Figure 1C). A threshold (|*J*_*ij*_| ≥ 0.1) was used to determine strongly connected edges. If we applied treatments multiple times, the energy landscape evolved as the iteration of treatment and restoration (Figure 2C).

The restoration process is an optimization procedure with reference to the stable pre-treatment state (Figure 2B) as shown in Equation 7. When the restoration process is saturated (no significant improvement in minimizing the functional distance by changing parameters), the saturated network becomes a new pre-treatment state for a new restoration step, i.e., ${A_{\infty }}^{r}=A^{p}$ (Figure 2C).

To implement the recovery process in Equation 7, we adopted the gradient ascent method, which is generally used to estimate the pairwise MEM model parameters from the experimental data by maximizing the log-likelihood (Watanabe et al., 2013, 2014a; Kang et al., 2017, 2019). To maximize the log-likelihood, model parameters, *H*_*i*_ and *J*_*ij*_, are updated iteratively according to differences between data-driven and model-driven expectations of activations and coactivations, as shown below.

(10) $$H_{i}\left(t+1\right)\leftarrow H_{i}\left(t\right)+\alpha_{g}\left(\text{log }\langle \sigma_{i}\rangle -\log\;{\langle \sigma_{i}\rangle }_{A}\right),$$

(11) $$J_{ij}\left(t+1\right)\leftarrow J_{ij}\left(t\right)+\alpha_{g}\left(\log\;\langle \sigma_{i}\sigma_{j}\rangle -\log\;{\langle \sigma_{i}\sigma_{j}\rangle }_{A}\right),$$

where α_*g*_ is a learning rate, 〈σ_*i*_〉 and 〈σ_*i*_σ_*j*_〉 are expectations of activations and coactivations of the brain regions evaluated using the empirical data. From the pairwise MEM parameter A, probability *p* (*V*_*k*_|*A*) for each state *V*_*k*_ can be derived using Equation 3, based on which the expected activations and coactivations of the brain regions are derived using the following equation.

(12) $$\begin{array}{l} {\langle \sigma_{i}\rangle }_{A}=\overset{2^{N}}{\underset{k=1}{\sum }}\sigma_{i}\left(V_{k}\right)p\left(V_{k}|A\right),\; \end{array}$$

(13) $$\begin{array}{l} {\langle \sigma_{i}\sigma_{j}\rangle }_{A}=\overset{2^{N}}{\underset{k=1}{\sum }}\sigma_{i}\sigma_{j}\left(V_{k}\right)p\left(V_{k}|A\right). \end{array}$$

If the dynamics of a pretreated system *A*^*p*^ follows the pairwise MEM (we assumed in this study), the expected activations 〈σ_*i*_〉 and coactivations 〈σ_*i*_σ_*j*_〉 of sufficiently large samples from the stabilized system *A*^*p*^, equal to the model-driven expectations of the activation ${\langle \sigma_{i}\rangle }_{A^{p}}\;$and coactivation ${\langle \sigma_{i}\sigma_{j}\rangle }_{A^{p}}\;$of the brain regions. Then, a recovery process can be explained as follows:

(14) $$\begin{array}{l} H_{i}\left(t+1\right)\leftarrow H_{i}\left(t\right)+\alpha_{g}\left(\log{\;\langle \sigma_{i}\rangle }_{A^{p}}-\log{\;\langle \sigma_{i}\rangle }_{A^{r}}\right), \end{array}$$

(15) $$\begin{array}{l} J_{ij}\left(t+1\right)\leftarrow J_{ij}\left(t\right)+\alpha_{g}\left(\log{\;\langle \sigma_{i}\sigma_{j}\rangle }_{A^{p}}-\log{\;\langle \sigma_{i}\sigma_{j}\rangle }_{A^{r}}\right),\\ \;A^{r}=\left\{H_{i},J_{ij}\right\}{|}_{i=1,\cdots \;,N,j=1,\cdots \;,N} \end{array}$$

The recovery is proceeded by adjusting the network parameters of neighbors ($A_{{\mathbb{R}}_{m}}^{t}$) of the treated node or edge *m*.

In general, a treatment is applied to a system when the recovery process is saturated for a sufficient time after each treatment. However, we also presented a simulation of treatment in the transient state before full saturation. We denote the transition state as the proportion of time relative to the time for full recovery. In this case, we used a grid search method to determine the optimal treatment time without waiting for full recovery, as well as the target region and its scale. In this situation, we denote the functional distance $D^{+}\;$ as a function of treatment timing *T*.

(16) $$A_{m}^{t*}=\arg\;\min\;D^{+}\left(A^{g},A^{s^{\prime }}|A_{m^{\prime }}^{s^{\prime }},T\right)$$

## Materials and Methods

### A Test System: the Subcortical Limbic Brain

**Figure 3:**
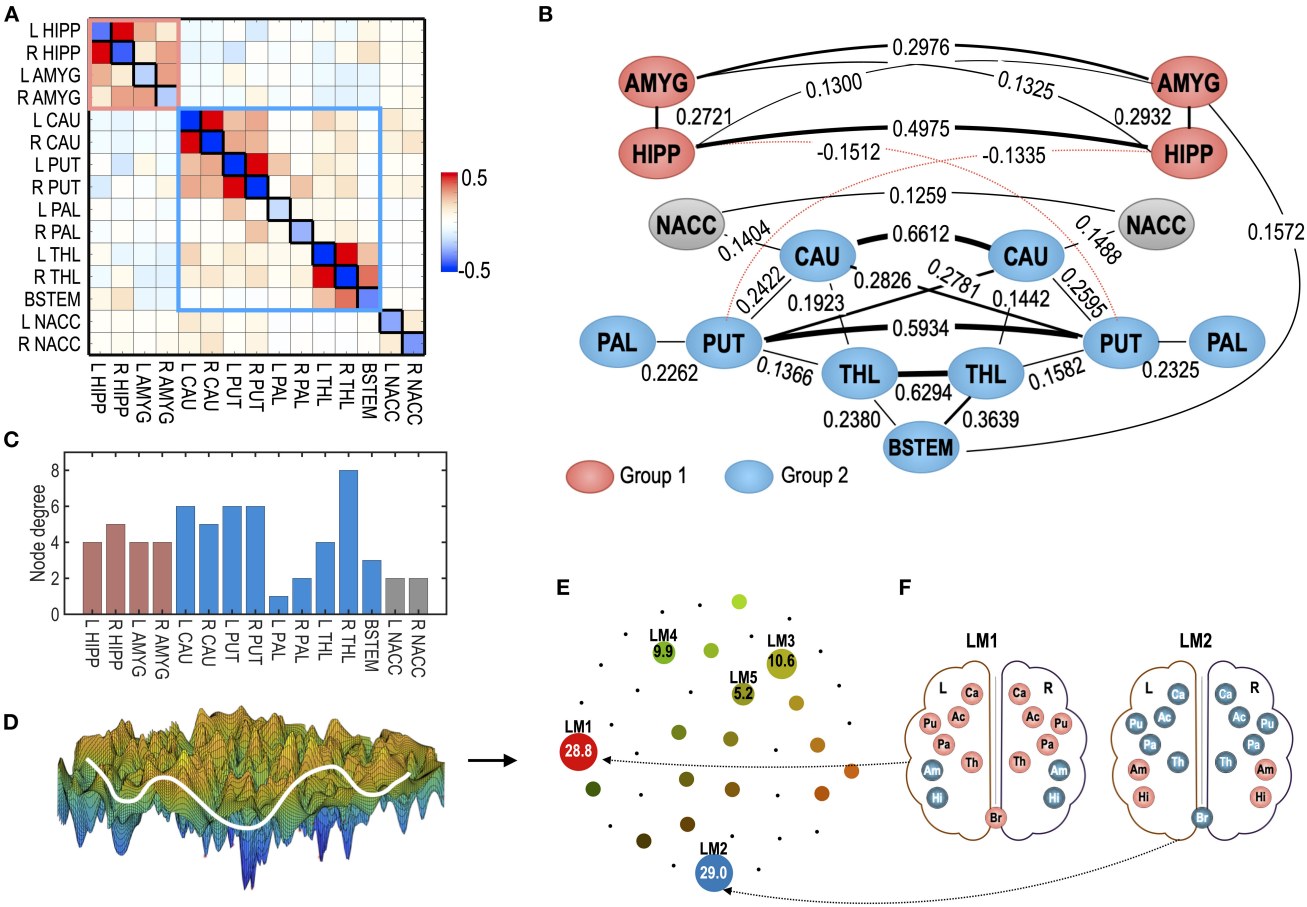
The human subcortical-limbic system used in the study as a test system. **(A)** MEM parameters of the baseline sensitivity parameter (*H*_*i*_) and pairwise interaction parameters (*J*_*ij*_) are displayed as diagonal and off-diagonal elements in the matrix. **(B)** The system is displayed in the interaction diagram. The pairwise interaction is displayed for |*J*_*ij*_| > 0.12. The thickness of the lines represents the strength of the pairwise interactions. The red dotted line represents negative pairwise interactions (*J*_*ij*_ < 0). Red and blue nodes represent ROIs that belong to modules 1 and 2. Modified from Kang et al. (2017). **(C)** Node degree of the subcortical-limbic system under a threshold |*J*_*ij*_| = 0.1 is shown. **(D)** A schematic illustration of the energy landscape of the subcortical-limbic system is displayed. **(E)** The energy landscape of the human subcortical system is represented in terms of local minima (LM) displayed in circles. The sizes of circles for LMs reflect the occupation time ratios (OCR) of LMs. The OCRs of LMs are displayed in percentiles. **(F)** Activation patterns corresponding to two major local minima (LM), i.e., LM1 and LM2, are displayed. The subcortical-limbic system includes the hippocampus (HIPP, Hi), amygdala (AMYG, Am), caudate (CAU, Ca), putamen (PUT, Pu), pallidum (PAL, Pa), thalamus (THL, Th), nucleus accumbens (NACC, Ac), and brainstem (BSTEM, Br). Red and blue colors represent activated and inactivated regions, respectively.

As a test system for optimal control, we reused the MEM for the subcortical human brain (Kang et al., 2017). Briefly, the system consists of 15 subcortical regions of interests (ROIs): the hippocampus (HIPP), amygdala (AMYG), caudate (CAU), putamen (PUT), pallidum (PAL), thalamus (THL), nucleus accumbens (NACC) of the left (L), and right (R) hemispheres, and the brainstem (BSTEM). The MEM parameters were estimated from the resting-state fMRI data of 470 participants in the HCP database (Van Essen et al., 2012). The estimated parameters are presented in Figures 3A,B. The subcortical-limbic system has highly symmetric interactions across hemispheres and appears to be modular. We sorted local minima (LMs) with their occupation time ratios and selected the five top local minima that have the highest occupation time ratios. Among 18 LMs, the five major LMs, i.e., LM1(8000), LM2(24769), LM3(32768), LM4(1), and LM5(25286), occupied 83.5% of all possible states (Figure 3E). The brain activation patterns for the two major local minima are displayed in Figure 3F. In this study, we set this system as a goal of the control for the virtual abnormal systems. By controlling the regional activity parameter *H*_i_ and pairwise interaction parameter *J*_*ij*_ (node and edge of the MEM parameters) of a virtual abnormal system, the abnormal system is expected to be guided to have dynamics of this goal system.

### Overview of Simulations

We conducted six simulation experiments to show the construct validity of the proposed framework. In simulation 1, we present an example to show the self-restoration process in the energy landscape of the brain network after treatment (or damage) in a region. In simulation 2, we present the effects of treatment on nodes and edges according to the number of neighbors to show the advantage of more neighboring edges in the restoration process. In simulation 3, we show the need for a self-restoration process in the control model by comparing treatment effects with and without considering the system's self-restoration. In simulation 4, we show the effects of repetitive treatment on each node of a source system to induce the desired dynamics. In simulation 5, we further control the timing of subsequent treatments before full recovery when treating a system. In simulation 6, we optimize the dissection of interhemispheric connectivity to simulate a corpus callosotomy for epilepsy surgery. All of these simulations were conducted to show the effects of the self-restoration process and how to treat the system to achieve the desired behaviors.

### Simulation 1: The System's Self-Restoration Process After Treatment

**Figure 4:**
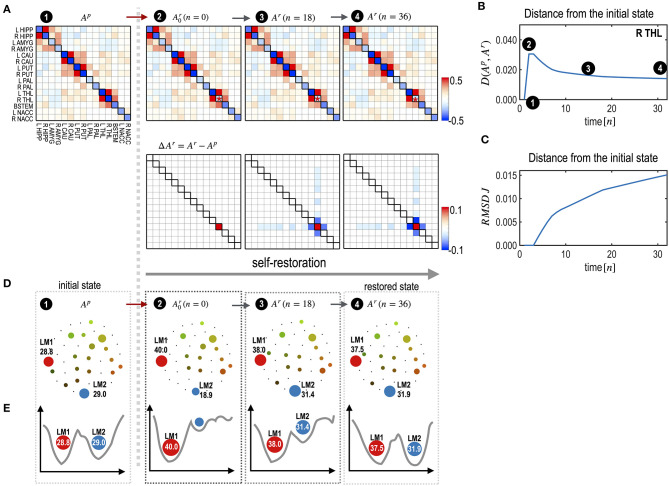
Explanation of the self-restoration process. **(A)** MEM parameters (and differences from the initial system) at the initial, treated (the time point *n* = 0), half-restored (*n* = 18), and fully restored (*n* = 36) states are displayed. **(B,C)** A distance curve (partial KL-divergence) **(B)** and an RMSD curve of J (interaction parameters) **(C)** from the pre-treatment state are displayed. Despite the increasing distance in parameter space, the distance of state dynamics from the pre-treatment state is reduced in the functional space. **(D)** Schematic diagrams for energy landscapes of the pre-treatment state (*A*^*p*^) and self-restored state (*A*^*r*^) are displayed along the time course. **(E)** Alterations in the energy landscapes following treatment and during the restoration process are explained in terms of the two major local minima LM1 and LM2. After restoration, the occupation time ratios of LM1 and LM2 become closer to those of the pretreated state.

To illustrate the self-restoration process, we presented a perturbation simulation of the right thalamus (R THL) by adding 0.5 to its *H*_*i*_ parameter. After this perturbation, the neighboring edges connected to the node were gradually reconfigured to generate state dynamics similar to those of the initial pre-treatment state (Figure 4A). For a treatment that induced a deviation of state dynamics from the pre-treatment state, the self-restoration procedure gradually moved the system toward the pre-treatment state, which induced a shorter functional distance D (partial KL-divergence) between the recovering and pre-treatment states (Figure 4B), and receded its parameters from those of the pre-treatment state (Figures 4B,C). Despite the increasing distance in the parameter space (RMSD, Figure 4C), the distance in the state dynamics from the pre-treatment state is reduced (D, Figure 4B). This is an example of degeneracy, which refers to the phenomenon where similar behaviors can be formulated using different networks.

Treatment of the right thalamus changed the energy landscape significantly from that of the pre-treatment state (Figure 4D); the OCR of LM1 increased from 28.8 to 40.0, and the OCR of LM2 decreased from 29.02 to 18.9. During self-restoration, this asymmetric energy landscape (between LM1 and LM2) gradually recovered (Figure 4D). In the final stage of the self-restoration, the OCRs of LM1 and LM2 were 37.5 and 31.9, similar to those of the initial system. Full recovery was not achieved in this system as it utilized only the limited resources of neighboring edges. Figure 4E shows the changes in the major two local minima (LM1 and LM4) along with the treatment and transient states in the recovery process. This suggests that the system recovers similar energy landscapes after recovery.

### Simulation 2: Region-Specific Self-Restoration Capacity

**Figure 5:**
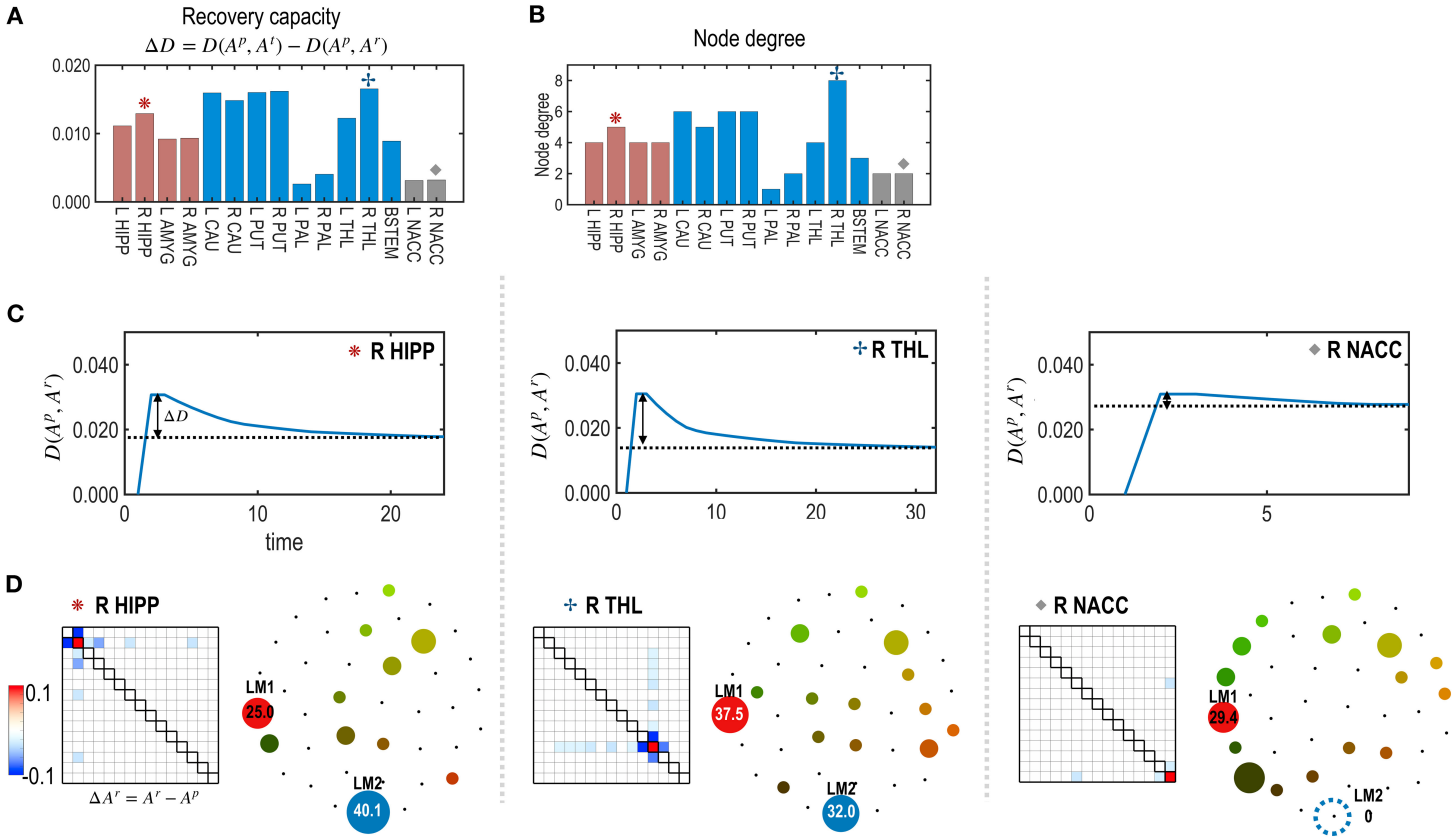
The recovery capacity at the nodes of the subcortical-limbic system. **(A)** Recovery capacity **D** is shown for each node. **(B)** The node degree plays as degrees of freedom, i.e., numbers of adjustable parameters for the recovery. **(C)** For the three representative nodes (R HIPP, R THR, and R NACC), recovery curves are displayed in terms of the functional distance (partial KL divergence) between the restoring state and the pre-treatment state. **(D)** Differences between parameters of the restored and pre-treatment states, and schematic energy landscapes at the final restoration state are displayed. The restoration at the R THL was most successful in terms of recovering the energy landscape of the pretreated state.

**Figure 6:**
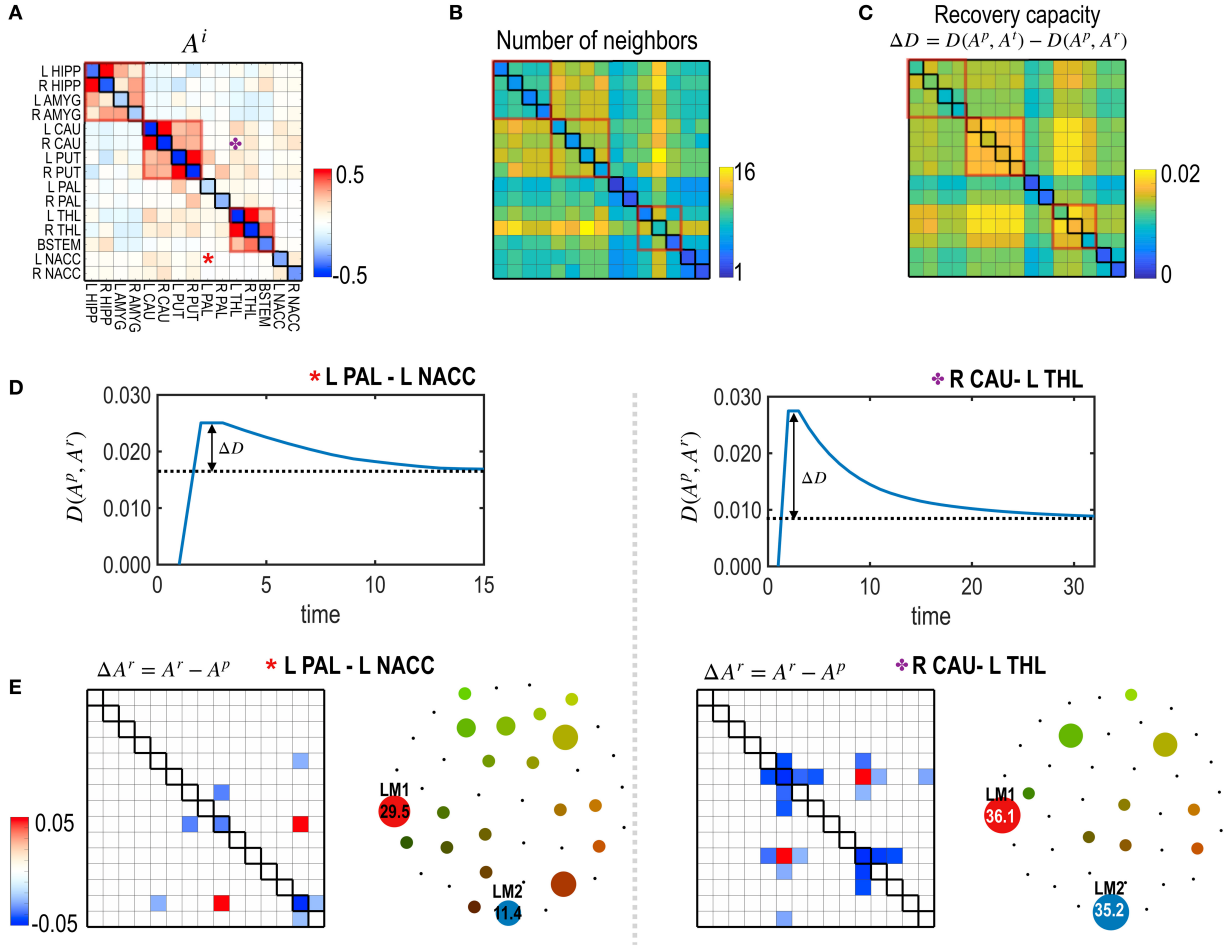
The recovery capacity at the edges of the subcortical-limbic system. **(A,B)** The model parameters **(A)** and the number of neighbors ℝ_**m**_ as degrees of freedom of the subcortical-limbic system **(B)** are presented. **(C)** Recovery capacities ΔD are shown for all the edges. Larger self-restoration occurred in the edges with high degrees of freedom (e.g., orange asterisk; edge between R CAU-L THL) than edges with lower degrees of freedom (e.g., red asterisk; edge between L PAL-L NACC). Two representative examples of treatments on the two edges are presented to show their differences in the recovery processes. **(D)** The distance curves (the partial KL-divergence) from the pretreated state are presented for the two representative edges; L PAL–L NACC (red asterisk) and R CAU–L THL (orange asterisk). **(E)** Differences in the MEM parameters of the recovered states from the pre-treatment state and the schematic energy landscapes of the final states for the recovered two edges are illustrated.

To test the node- or edge-specific recovery capacity, we evaluated the self-restoration process after treating each node (Figure 5) and each edge (Figure 6) one by one. The degree of freedom was defined by the number of neighboring edges that participated in the self-restoration process. In the node's treatment, the neighboring edges were strongly (threshold (|*J*_*ij*_| ≥ 0.1) connected with the treated node (Figure 1B). In the treatment of an edge, neighboring nodes connected to the treated edge and strongly connected edges connected to these neighboring nodes were considered to participate in the self-restoration process (Figure 1C).

Figure 5 presents the node-specific restoration process in the real subcortical-limbic system. Nodes with diverse node degrees (Figure 5B) have different recovery capacities (Figure 5A) and recovery curves (Figure 5C). The finally recovered network parameters and energy landscapes differed from each other (Figures 5C,D). The recovery capacity depended highly on the number of neighbors (or node degrees); nodes with more neighbors had a higher recovery capacity. In this case, the node degree acted as a degree of freedom of the system. This was also found in the treatment of edges shown in Figure 6. In this case, the number of neighboring edges of the system again explained the recovery capacity. The edges with a higher number of neighboring edges (size of neighbors ℝ_*m*_ in Figure 6B) showed better restoration (Figure 6C). For example, greater self-restoration occurred after treatment in the edge between R CAU and L THL compared to the edge between L PAL and L NACC (Figure 6D). The latter utilizes adjustments of more edges than the former one in the self-restoration (Figure 6E).

### Simulation 3. Effects of the Self-Restoration Process in Controlling the Brain System

**Figure 7:**
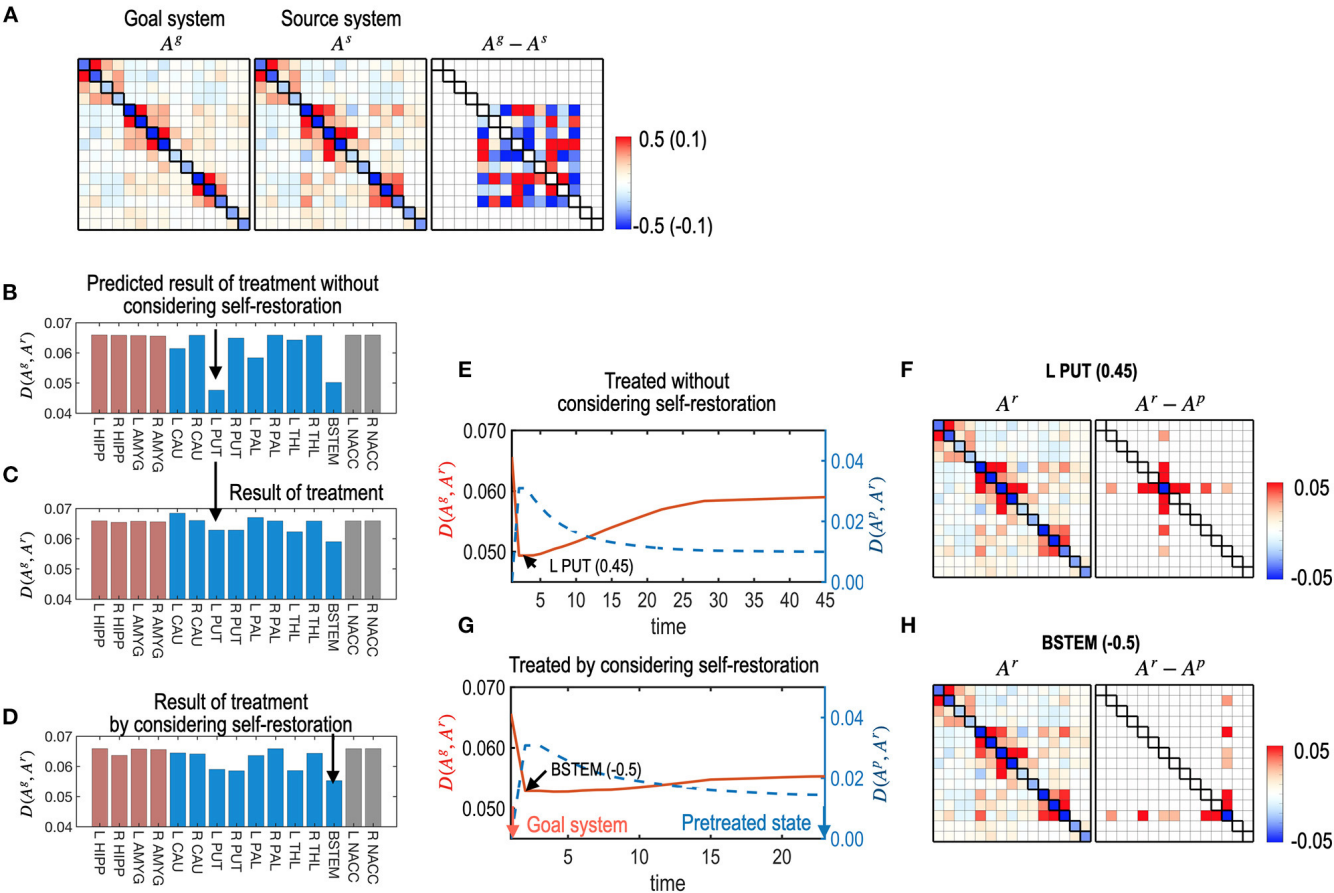
Treatments with and without considerations of the self-restoration. **(A)** Model parameters of a virtual (source) system and the subcortical-limbic system as a goal system, and the difference between the goal and the source systems are displayed. The virtual system is generated by adding a noise (**A**^**g**^**−A**^**s**^) to the subcortical-limbic system. **(B)** The distance between the goal state and the predicted final state, **D** (**A**^**g**^, **A**^**r**^), after treatment at each node without considering the self-restoration, was minimal for the treatment on the L PUT. **(C)** The final results of treatments are displayed in **(B)**, which show longer distances from the goal state than predicted due to the restoration process. For example, the treatment on node L PUT with strength 0.45 results in distance 0.074, which was expected 0.055 in the prediction without considering the self-restoration process. **(D)** The final distance for each node treated by considering self-restoration is displayed. In this case, BSTEM was selected as an optimal treatment node with −0.5 strength. **(E–H)** The treatment and restoration curves in terms of the distance from the goal system (red) and the pretreated state (blue) for the treatment on L PUT with strength of 0.45 **(E)** and for the treatment on BSTEM with strength of −0.5 **(G)** are displayed. MEM parameters (and differences from the pretreated state) at the final state are displayed for the treatment on L PUT **(F)** and for the treatment on BSTEM **(H)**.

We simulated treatments with and without considering the self-restorative properties of the system. In this study, we generated a virtual system by adding a Gaussian random noise ~*N* (0, 0.1) to the parameters of the human subcortical-limbic system presented in Figure 3. We considered the virtual system as a source system and the human subcortical system as a goal system (Figure 7A).

For each node, the best strength of treatment (|α|) was identified using a grid search method among a set of α, 0.05, 0.10, 0.15, …, and 0.5. We selected the best treatment strength that minimizes the functional distance between the final restoration state and goal state based on Equations 8, 9. Figure 7B shows the expected treatment effects without considering the restoration process, while Figure 7C shows the actual treatment results for the self-restorative system. Discrepancies between the expected and treatment effects occurred when the self-restoration process was not considered. In contrast, when the recovery process was considered, we obtained increased treatment effects (Figure 7D), with functional distances from the goal system shorter than that of treatments without considering the system's recovery process (Figure 7C). Optimal nodes differed according to how the nodes were chosen with or without a self-restorative model. When we determine the optimal node and its treatment strength to treat without considering the recovery process, an optimal treatment target was chosen in the left putamen (L PUT) with a treatment strength of 0.45 (Figure 7B) but the optimal treatment did not effectively change the system to the desired goal after restoration (Figures 7C,E). When we consider the restoration's effects, the best treatment was selected on BSTEM with−0.5 (Figure 7D). For this treatment, functional distance from the desired state decreased right after treatment, followed by an increase during the restoration process (Figure 7G). The final treatment effects by considering the restorative process are better in this treatment than the treatment without considering restoration (Figure 7E).

### Simulation 4. Repetitive Treatment at a Single Node

**Figure 8:**
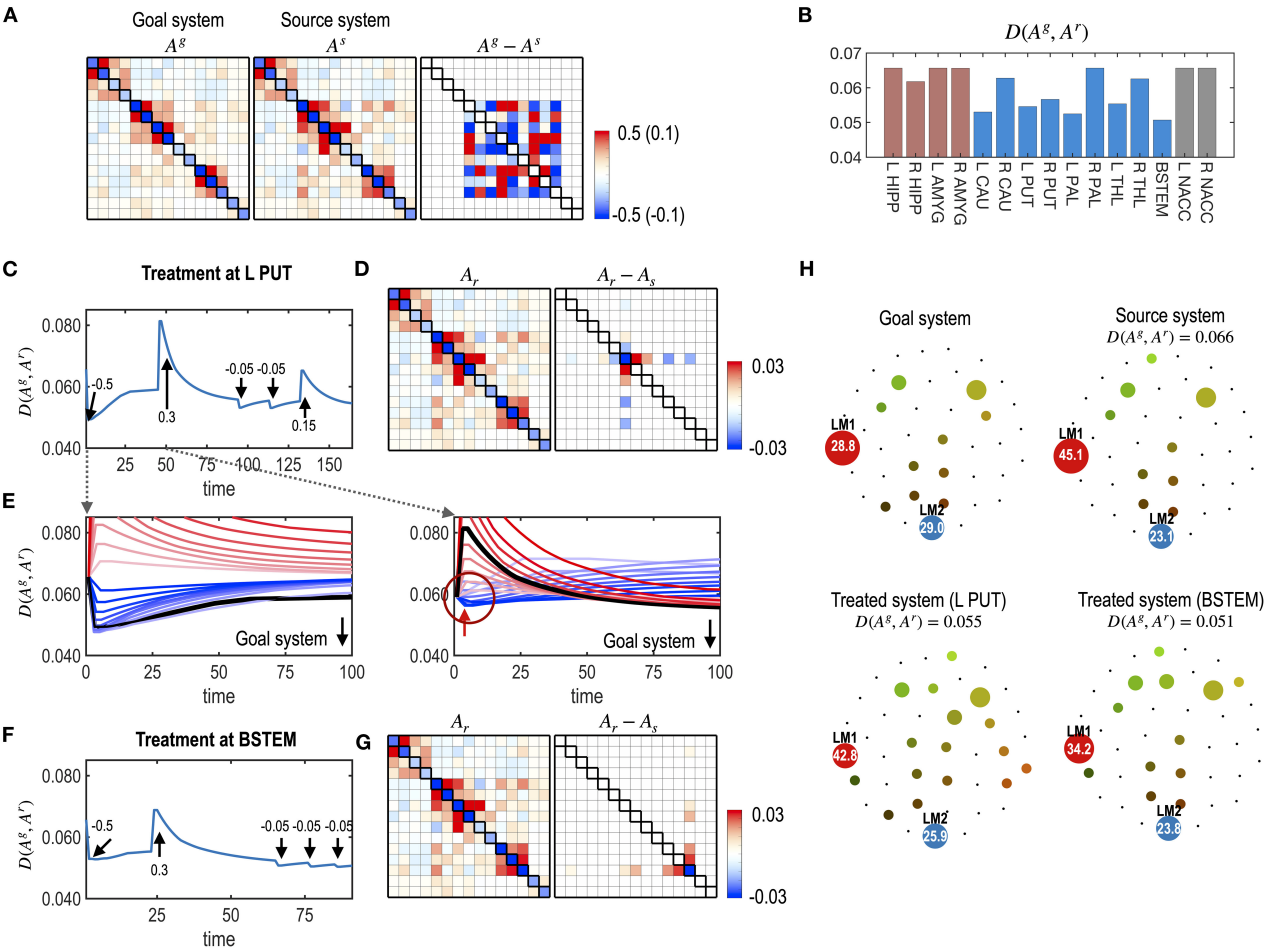
Multiple treatments for the nodes of a system. **(A)** MEM parameters of the virtual source and goal systems, and their differences are displayed. **(B)** The distances (partial KL divergence) between the final states and the desired state are displayed for each treatment. The optimal treatment was chosen at the BSTEM. Results of the treatment at L PUT **(C–E)** and BSTEM **(F,G)** are shown. **(C)** A treatment curve (distance between the transient state and the desired state) with a treatment strength at each treatment (arrows) is displayed. **(D)** MEM model of the final state and its difference from the pre-treated state are shown. **(E)** Restoration curves with potential strengths of the treatment are displayed for the first and second treatments. In the first treatment, the treatment strength that induces the black line was chosen as the optimal strength since it finally becomes closest to the desired state after saturation. After a treatment, it gets close to the desired state and then recedes slightly from it. In the second treatment, some treatment strengths make the system closer to the desired state at the early stage of the treatment (blue lines in the circle). However, those curves eventually diverge from the goal system. The optimal treatment strength induced a curve colored in black, which initially deviates from the goal system but eventually comes closer to the desired state than any other treatment strengths. For clarity, we scaled the restoration time for each treatment. **(F,G)** A treatment curve at the BSTEM **(F)** and its final MEM parameter and its differences from the pre-treated state **(G)** are shown. **(H)** Energy landscapes of the goal, the virtual source, and final systems after treatment at L PUT and BSTEM are displayed.

In the clinic, most treatments are repetitive, particularly concerning medications. We simulated repetitive treatments without changing the treated node. In the repetitive treatment simulation, the subsequent treatment was applied after the effects of the previous treatment had become saturated (i.e., reached an equilibrium state). Figure 8 shows the results of the repetitive treatment at each node. Compared to the single treatment shown in Figure 7D, repetitive treatments with a sequence of different strengths (Figures 8C,F) made the system closer to the desired goal (Figure 8B). After repetitive treatment, the treated system's final energy landscape gets closer to that of the target goal system (Figure 8H).

In Figure 8E, the state change due to the second treatment suggests the non-linear nature of the treatment vs. the behavioral response (dynamics). The optimal treatment was not always chosen to minimize the distance to the goal system in the early stages of the time curves, as shown in the first treatment effect (Figure 8E). Instead, the optimal treatment made the system deviate from the desired goal system but eventually get closer to the desired system than other treatments that are initially effective but finally ineffective (Figure 8E).

### Simulation 5. Repetitive Treatment at a Single Node With Flexible Timing

**Figure 9:**
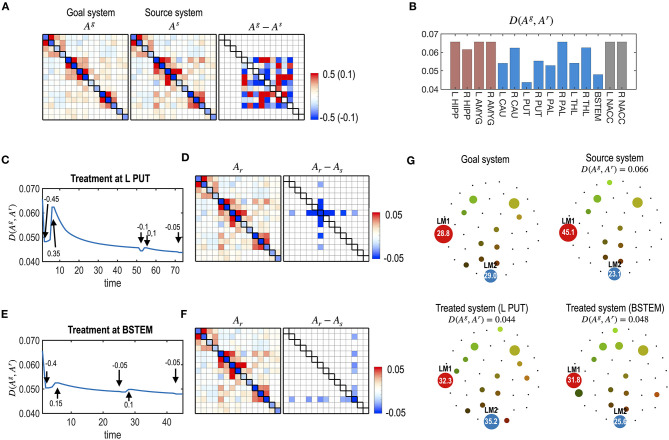
Repetitive treatments with flexible treatment timing. **(A)** The same model in Figure 8 is used for the current simulation. **(B)** The distance between the goal and final states, **D** (**A**^**g**^, **A**^**r**^), from multiple treatments with flexible timing of treatment is evaluated for each node. The best node for the multiple treatments was the L PUT. **(C–F)** Results of the treatments at L PUT **(C,D)** and at BSTEM **(E,F)** are displayed. **(C,E)** Treatment curves (the distance between the transient states and the goal system) are displayed for L PUT and BSTEM. The arrows indicate the timing of each treatment, and the values are the strengths of the treatment. **(D,F)** MEM parameters of the final system and its difference from the initial system are shown. **(G)** Energy landscapes of the goal system, the virtual source system, and final systems after treatment at the L PUT and BSTEM are displayed.

Most previous studies did not consider the timing of the treatment under the dynamically responding brain. When a treatment is applied, the brain gradually recovers and transitions to an equilibrium state. Considering the restoration process, one may apply the subsequent treatment at transient states without waiting for the equilibrium state. We simulated optimal repetitive treatment by optimizing the timing of subsequent treatments. In this simulation, we used the same simulation setting as simulation 3, except for the flexible timing of the treatment. Compared to simulation 4, we explored the best strength of the treatment and the best timing of subsequent treatments for each node. As shown in Figure 9, the treatment with the best timing increased the treatment effect compared to treatment after full recovery for each treatment (Figure 8).

### Simulation 6. Optimal Removal of Edges

**Figure 10:**
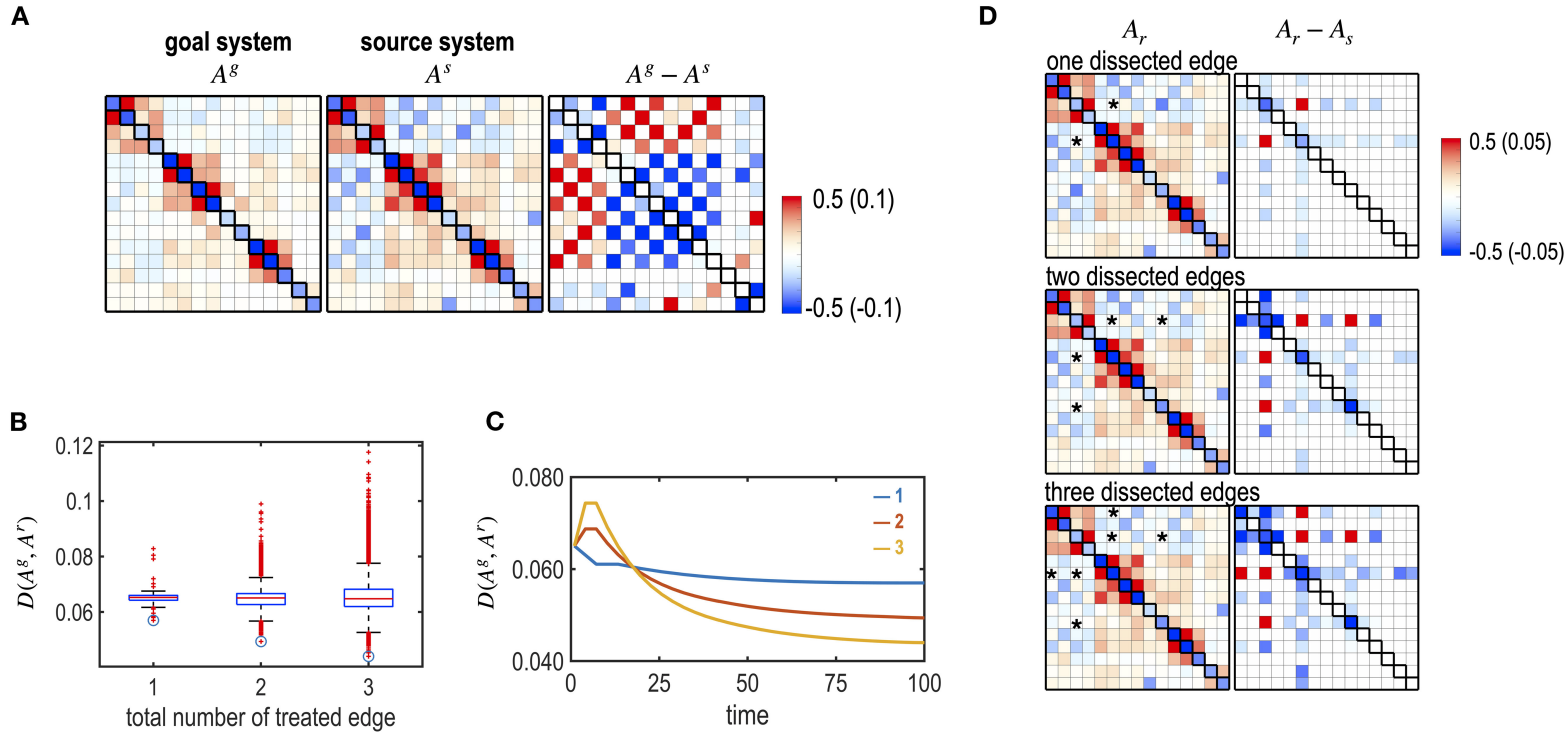
Optimal dissection of edges, simulating a corpus callosotomy for epilepsy surgery. **(A)** MEM parameters of the virtual (abnormal source) system and the goal system (subcortical-limbic system), and their differences are displayed. The virtual source system was generated by increasing the strengths of the connectivity between the left and right hemispheres. **(B)** The box graph shows the final distance between the goal and the treated system, **D** (**A**^**g**^, **A**^**r**^) for all the combinations of dissected edges by dissecting one, two, and three inter-hemispheric edges. Optimally treated results for each number of edges are denoted with circles. **(C)** Treatment curves (the distance between the transient state and the goal system) are displayed for the optimally chosen edges among the one (blue line), two (red line), and three (yellow line) edge combinations of dissection. **(D)** MEM parameters of the final systems after optimal treatments with different numbers of dissected edges are displayed with their differences from the initial source system.

We simulated dissections of interhemispheric connections to imitate a corpus callosotomy for epilepsy surgery. We generated an abnormal brain that has stronger connections between the left and right hemispheres (Figure 10A). This was performed by increasing positive inter-hemispheric connectivity and decreasing the negative interhemispheric connectivity of the subcortical-limbic system by adding a Gaussian noise ~*N* (0, 0.1) according to the polarity of the initial connectivity. We tested the optimal treatment strategy for different numbers and targets of interhemispheric edges (one, two, and three) to be dissected (Figures 10B,C). For each number of edges (49 single interhemispheric edges, i.e., left 7 × right 7, 2,352 combinations for two edges, and 110,544 combinations for three edges), we evaluated the best edges to remove. For the source brain, dissection of LAMYG-RCAU (one edge); LAMYG-RCAU and LAMYG- RPAL (two edges); LAMYG-RCAU, LAMYG- RPAL, and LHIPP- RCAU (three edges) were the best combinations to decrease the functional distance from the goal system (Figure 10D).

## Discussion

Although brain control has garnered increasing interest, brain control research has mainly been conducted based on theoretical and computational models as the practical control of the brain has many challenges due to the complexity of the brain and ethical issues. Several computational models to control the brain network have been proposed to characterize the graph-theoretic properties of the system (Tang et al., 2017; Lee et al., 2019; Stiso et al., 2019; Karrer et al., 2020) or a purpose of predicting outcomes after treatment (Falcon et al., 2016; Jirsa et al., 2017; Proix et al., 2017; An et al., 2019; Olmi et al., 2019). These previous studies have assumed the brain as a dynamic system, immediately responding to the incoming treatment. However, the system's self-restoration process after the cessation of the treatment has not been fully considered, without which the brain control may not be optimal. Compared to the brain circuit's various dynamic state equations, the formulation of the self-restoration process has been rarely researched.

To account for the effect of the restoration process on brain control, we propose a formulation of the brain's recovery process that drives the system to perform the function before treatment. The driving force of this self-restoration process is based on the free-energy principle (Friston et al., 2006; Friston, 2010) over a non-linear complex system, with degeneracy in terms of generating the same behaviors from diverse network configurations. According to the free-energy principle (Friston et al., 2006; Friston, 2010), the network is configured to respond or predict the environment's statistical demands, making the system energy-efficient. As long as the environment's statistics do not change, the treated or partially lesioned system may well-adjust its remaining subnetwork (neighbors of the treated node) to satisfy those demands. Since the altered (treated) node cannot participate in the organized work of the subnetwork at the same performance level as a pre-treatment state, the system tries to compensate for the role of the altered node by reorganizing interactions with its neighbors. This restoration process in the brain can be called a type of optimization process in that the system tries to adjust itself and gradually approximates the desired functionality of the pre-treatment state by interaction with the environment under biological constraints.

The other central concept of the current study is the redundant nature of the non-linear brain (Glassman, 1987; Edelman and Gally, 2001). A complex system has degeneracy, i.e., the same or similar functions (behavior) can be achieved using different configurations of networks (or connectivity). Since it is complicated to restore all connectivity after damage, optimal control utilizes non-linearity between networks and behaviors by reconfiguring networks among neighbors within limited ranges to approximate the goal system dynamics. In this non-linear relationship, the closeness in the system parameters (e.g., connectivity) does not necessarily indicate closeness in behaviors. Instead of matching network connectivity, the current framework fits behaviors (i.e., microstate dynamics) by modulating a smaller number of network parameters. This is possible as the brain is a complex non-linear system from which non-linear microstate dynamics emerge.

The non-linear microstate dynamics of a brain are represented in the microstate's energy landscape, the microstate of which is often defined by a distributed activity pattern over the temporal scale of a second. Energy landscape analysis has been applied to explore dynamics in large-scale functional brain networks, such as the default mode and pre-frontal networks, on resting-state fMRI (Watanabe et al., 2013, 2014a; Kang et al., 2017, 2019) and in sleep (Watanabe et al., 2014b). In our previous study (Kang et al., 2017), the energy landscape analysis revealed that the subcortical brain at rest exhibits the maximal number of stable states and small sets of stable states account for most of the occupation time. Furthermore, a graph theory analysis of the energy landscape revealed a hub-like state transition organization embedded in the resting-state human brain (Kang et al., 2019). The energy landscape of brain states is governed by a set of network parameters in the pairwise MEM, upon which treatment is imposed.

The brain control extends the energy landscape concept at the temporal scale of a second (microstates) to the energy landscape over a more extended period. Over a longer period of years, a brain can be considered in a network state of the macroscopic energy landscape. For example, the brain develops from one network state (a set of network parameters) to another (another set of network parameters). A network state in the macroscopic energy landscape is defined by a network parameter, which differs from the definition of a microstate in the microscopic energy landscape by a distributed activity pattern. In this respect, the brain control problem is to choose an optimal way to guide a brain network to a desired network along the macroscopic energy landscape.

We assumed that the restoration process is conducted by the cooperative activity among neural populations within the brain network, which tries to generate similar functionality established before treatment. In this process, the brain network's modularity, an essential property to protect against damage to a complex brain (Park and Friston, 2013; Sporns and Betzel, 2016), would be crucial for the recovery. By rearranging resources within a module (e.g., altering connectivity within a biological range), the modularity actuates the system's reorganization to construct a similar behavior. There is plentiful evidence of modular-based reorganization in brain diseases (Balenzuela et al., 2010; Chen et al., 2013; Siegel et al., 2018). In this study, nodes with more (stronger) connections (functional neighbors) play a more efficient role in restoration than nodes with fewer connections. Since the current test system has a relatively small network size (nodes = 15), we did not restrict the neighbors within a module but functionally close regions (Figure 6). Even though not strictly the same as the modularity concept in systems science, the functional neighbors work as a functional module in terms of cooperation within the module. Consideration of neighbors restricted within a functional module and within an anatomical limit of a larger network would be more realistic in modeling the recovery process.

Clinical treatment is generally exerted on the brain multiple times. After treatment, e.g., antipsychotic medication, clinicians wait to stabilize the brain to avoid transient states. However, one may consider applying subsequent treatment before stabilization. Until fully stabilized, the system has multiple transient states for network parameters. Some transient states may be more efficient in achieving a desired goal than the stabilized state. However, the transient state may be unstable, and finding an optimal strategy may be unpredictable. In the current study, we showed a possibility to optimize the right timing without waiting for complete stabilization when we have a model for self-restorative process.

The current framework as computational modeling takes advantage of prediction capacity by simulation. It is theoretically possible that some treatment parameters may lead the treated system unstable, generating abnormal functionality. The self-restoration process may also cause the malformation of the function. By evaluating treatment outcomes for all possible ranges of parameters, we may check unstable points before deciding the treatment. The model-based prediction could also be used in evaluating the treatment effect due to noise in the restoration process as a type of Monte-Carlo simulation (See the Supplementary Material). As the noise effects differ across brain regions, one may choose a reliable target that is less sensitive to noise in the restoration process. The treatment could benefit from evaluating the treatment outcomes with noise in any parameters or any updating rules besides the restoration process. This Monte-Carlo simulation may complement the limitation of the current deterministic approach. We used a simple deterministic model and its solver for the control problem to explain the basic framework of brain control and show the current framework's construct validity. More sophisticated models based on more advanced control theory methods, such as a stochastic model proposed by Todorov (2009), could be further researched.

In this study, we showed the construct validity of the proposed framework using various simulations to consider the clinical environment. A simulation suggests that the optimal brain control should include the system's self-restoration process, without which a (so called optimal) treatment is not optimal. Using simulation, we also proposed how to control the self-restoration process by choosing the optimal target region and treatment strength. We then presented simulations for optimizing repetitive treatments and the optimal timing of treatment. We found that some treatment choices led to a degraded performance at an early stage but eventually showed a better treatment effect (Figure 8). This is a typical example of the non-linear property of the self-restoration system that should be considered in optimal control. All of these simulations suggest the plausibility and rationale of the proposed brain control framework.

The current study is theoretical, and we acknowledge all possible limitations of the theoretical framework. The current brain control framework will be more practical when we know more about the system's reorganization mechanisms. Empirical experiments and validation are most demanding. Determining the means of achieving the desired treatment level at the right target for each treatment is one of the fundamental challenges. The details of the restoration process require extensive research and experiments. There exist many challenges before brain control can be applied to actual experiments. However, the current conceptual framework with the self-restorative process in the treatment is highly needed in clinical practices, which calls for personalized treatments based on individualized self-restoration systems and basic neuroscience to understand how the brain works.

In summary, we propose an optimal brain control framework by introducing self-restoration processes in the brain after treatment. Simulation results showing the responses and movement of a source system toward the desired system in diverse testing sets suggest the framework's plausibility in optimal brain control within a restricted treatment environment. Although further research with experimental data should be conducted, we believe the proposed computational framework would help attain optimal brain control of the dynamic self-restorative brain after treatment.

## Data Availability Statement

The MATLAB codes used in the current study are available by request or online from a public drive, http://bit.ly/monet-optimal-control-MEM.

## Author Contributions

H-JP developed the idea and JK performed experiments. Both wrote the manuscript.

## Conflict of Interest

The authors declare that the research was conducted in the absence of any commercial or financial relationships that could be construed as a potential conflict of interest.
