# Supplementary material for: A Computational Framework for Controlling the Self-Restorative Brain Based on the Free Energy and Degeneracy Principles
Source: Front Comput Neurosci. 2021 Apr 14;15:590019. doi: 10.3389/fncom.2021.590019 (PMC8079648; doi:10.3389/fncom.2021.590019)
Supplement: Supplementary file 1 [file Table_1.DOCX]

Supplementary Material

A computational framework for controlling the self-restorative brain based on the free energy and degeneracy principles

Hae-Jeong Park^1,2,3,4,*^, Jiyoung Kang^1,2*^

^1^Center for Systems and Translational Brain Science, Institute of Human Complexity and Systems Science, Yonsei University, Seoul, Republic of Korea

^2^Department of Nuclear Medicine, Department of Psychiatry, Yonsei University College of Medicine, Seoul, Republic of Korea

^3^Graduate School of Medical Science, Brain Korea 21 Project, Yonsei University College of Medicine, Seoul, Republic of Korea

^4^Department of Cognitive Science, Yonsei University, Seoul, Republic of Korea

*These authors contributed equally

*** Correspondence:**

Hae-Jeong Park, PhD
Email: parkhj@yonsei.ac.kr

Jiyoung Kang, PhD
Email: jykang@yuhs.ac

# Evaluation of noise effects of the recovery process

To evaluate the present scheme's dependency on the noise in the recovery process, we performed a Monte-Carlo simulation based on Simulation 3 of the Method section with an additional noise term in the self-restoration process. The activation and coactivation model parameters, $H_{i}$ and $J_{ij}$, are updated iteratively according to differences between data-driven and model-driven expectations of activations and coactivations with a noise $\varepsilon$. The equations for updating model parameters are modified as below.

$$H_{i}\left( t+1 \right)\leftarrow H_{i}\left( t \right)+\alpha_{g}\left( \log\left\langle\sigma_{i} \right\rangle-\log\left\langle\sigma_{i} \right\rangle_{A} \right)+\varepsilon, (10)$$

$$J_{ij}\left( t+1 \right)\leftarrow J_{ij}\left( t \right)+\alpha_{g}\left( \log\left\langle\sigma_{i}\sigma_{j} \right\rangle-\log\left\langle\sigma_{i}\sigma_{j} \right\rangle_{A} \right)+\varepsilon, (11)$$

where $\alpha_{g}$ is a learning rate, $\left\langle\sigma_{i} \right\rangle$ and $\left\langle\sigma_{i}\sigma_{j} \right\rangle$ are expectations of activations and coactivations of the brain regions evaluated using the empirical data. We generated noise $\varepsilon$ that follows a Gaussian distribution *N*(0, $\sigma^{2}$) for each iteration.

To focus on the noise's effects, we fixed the treatment strength as the best strength found in Simulation 3 without noise. We performed simulations with different noise levels, $\sigma=0.1, 0.2,$ and 0.3. For each noise level, a total of 30 simulations was conducted.

The results show that the noise effects differed across brain regions. For example, the recovery error compared to the desired target state, measured by $D\left( A^{g},A^{r} \right)$, increased considerably due to noise in the recovery process at some nodes (L CAU, R CAU, L THL, R THL, and R PUT). The uncertainty of the treatment effect due to the recovery process's uncertainty also increased significantly in those regions. This result suggests the importance of evaluating treatment outcomes due to noises in the recovery process. One may choose a treatment target that is robust to noise rather than highly sensitive to noise in the restoration process. The current computational modeling framework provides an easy way to evaluate treatment reliability due to the noise in the recovery, as exampled in this simulation.


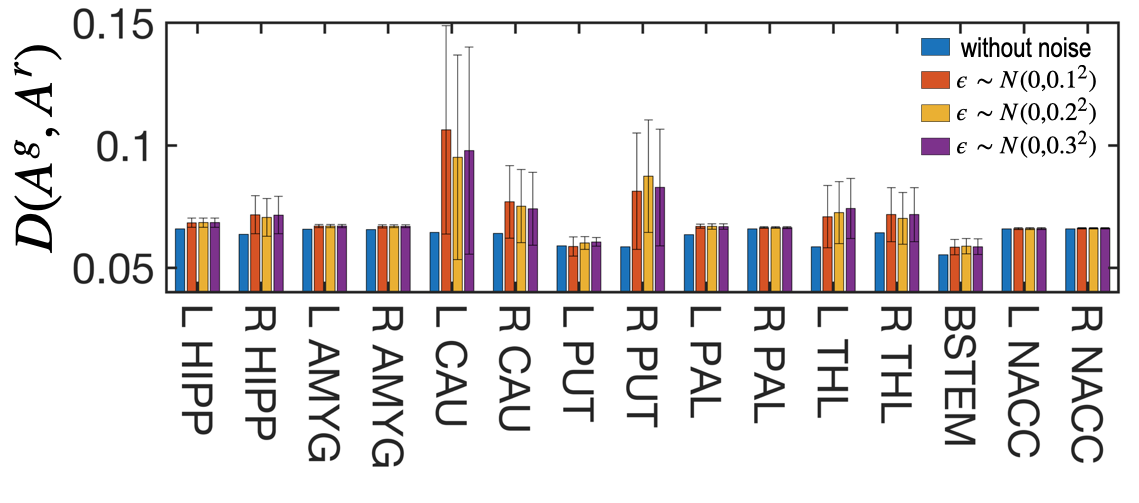


**Supplementary Figure 1.** Treatment outcomes according to noise in the self-restoration. Treatment errors, i.e., the averaged distances between the goal state and the predicted final state, $D\left( A^{g},A^{r} \right)$, were evaluated for the Gaussian noise with four different standard deviations, 0 (without noise, blue), 0.1 (red), 0.2 (yellow), and 0.3 (purple). For each noise level, a total of 30 simulations was conducted. The mean and standard deviation (error bars) are presented.
